# Structural features within the NORAD long noncoding RNA underlie efficient repression of Pumilio activity

**DOI:** 10.1038/s41594-024-01393-5

**Published:** 2024-09-26

**Authors:** Svetlana Farberov, Omer Ziv, Jian You Lau, Rotem Ben-Tov Perry, Yoav Lubelsky, Eric Miska, Grzegorz Kudla, Igor Ulitsky

**Affiliations:** 1 Department of Immunology and Regenerative Biology and Department of Molecular Neuroscience, Weizmann Institute of Science, Rehovot, Israel; 2 Wellcome Trust/Cancer Research UK Gurdon Institute, University of Cambridge, Cambridge, UK; 3 MRC Human Genetics Unit, University of Edinburgh, Edinburgh, UK

## Abstract

Long non-coding RNAs (lncRNAs) are increasingly appreciated for their important functions in mammalian cells. However, how their functional capacities are encoded in their sequences and manifested in their structures remains largely unknown. Some lncRNAs bind to and modulate the availability of RNA-binding proteins, but the structural principles that underlie this mode of regulation are unknown. The NORAD lncRNA is a known decoy for Pumilio proteins, which modulate the translation and stability of hundreds of mRNAs, and, consequently, a regulator of genomic stability and aging. We probed the RNA structure and long-range RNA-RNA interactions formed by human NORAD inside cells under different stressful conditions. We discovered a highly modular structure consisting of well-defined domains that contribute independently to NORAD function. Following arsenite stress, most structural domains undergo relaxation and form interactions with other RNAs that are targeted to stress granules. We further revealed a unique structural organization that spatially clusters the multiple Pumilio binding sites along NORAD and consequently contributes to the de-repression of Pumilio targets. We then applied these structural principles to design an effective artificial decoy for the let-7 miRNA. Our work demonstrates how the sequence of a lncRNA spatially clusters its function into separated domains and how structural principles can be employed for the rational design of lncRNAs with desired activities.

## Introduction

Mammalian genomes are pervasively transcribed, with tens of thousands of unique loci producing long RNA molecules that do not serve as templates for the production of functional proteins. These RNAs, which are collectively called long non-coding RNAs (lncRNAs) ^1^, closely resemble mRNAs on the molecular level: they are capped, polyadenylated, and usually spliced. Compared to protein-coding genes (PCGs), lncRNAs as a group are somewhat shorter, expressed at lower levels, and are more tissue-specific ^2^. Whereas the catalogs of lncRNAs in mammals are well annotated, their modes of action remain largely obscure. The low abundance and large size of the lncRNA molecules make it difficult to apply the same biochemical approaches used for elucidating the molecular mechanisms of other classes of RNAs.

While most of the functionally characterized lncRNAs act in the nucleus, many other lncRNAs accumulate in the cytosol ^2^ and plausibly act in post-transcriptional regulation. Specifically, modulation of the activity of RNA binding proteins (RBPs) or small RNAs by lncRNAs via competition for binding is one of the most commonly suggested modes of action for lncRNAs to date. The molecularly indistinguishable characteristics of mRNAs and lncRNAs make the latter effective decoys in theory. However, there are substantial doubts regarding the feasibility of the direct competition model in light of the low expression of lncRNAs compared to that of RBPs, and the consequently relatively few RBP binding sites offered by any lncRNA gene compared to the sites found throughout the transcriptome ^3^. Understanding the features that turn lncRNAs into effective decoys is crucial for evaluating how common this mode of action is and for designing synthetic RNAs capable of competing with the activity of specific RBPs.

The NORAD lncRNA is one of the most abundant and conserved lncRNAs in mammalian cells ^4–6^. NORAD presence is required to prevent chromosome instability in HCT 116 cells ^4,6,7^ as well as prevent premature aging in mice ^8^. Additional consequences of loss of NORAD were described in endothelial cells ^9,10^ and in cancer cells ^11^. NORAD accumulates to hundreds or even thousands of copies per cell, mostly in the cytoplasm ^4,5,7,12^, although nuclear localization and activity were also reported ^6^. NORAD contains ~20 Pumilio Recognition Elements (PREs), which are binding sites for PUM1/2, two members of the Pumilio family of RBPs. PUM1/2 post-transcriptionally repress gene expression, and modulation of NORAD expression levels results in a corresponding transcriptome-wide change in the abundance of Pumilio targets ^4,5,8,13^. While the total number of sites offered by NORAD is substantially higher than that offered by any other single gene and is comparable to the total number of PUM1/2 protein molecules in a human cell, it is still small compared to the total number of binding sites offered by all other Pumilio targets combined ^14^. The formation of phase-separated NORAD-Pumilio (NP) bodies was recently shown to enable a more efficient competition for Pumilio binding by NORAD ^13^.

Little is known about the functionality of the non-PRE regions in NORAD. We recently used a massively parallel RNA assay to determine sequences within NORAD, mostly found near its 5’ end, which are sufficient for effective NXF1-dependent export of an intronless RNA ^15^, and a recent study has shown that this region binds RBM33 which is required for NORAD export to the cytoplasm ^16^. Both the PREs and non-PRE regions within NORAD were recently shown to be required for effective NORAD recruitment into stress granules upon metabolic stress ^12^. Additionally, a sequence in the 5’ region of NORAD was shown to be associated with RBMX protein and to be required for genome integrity ^6^. We found that the sequence of NORAD contains 12 sequence-similar “NORAD Repeat Units” (NRUs) ^5,17^. The Mendell lab recently showed that mutating all the PREs in NORAD is sufficient for abolishing its ability to prevent chromosome number instability in HCT116 cells and that a sequence containing only NRUs 7 and 8 is sufficient for this activity ^7^ (NRUs 7+8 correspond to ND4 in the notations used by the Mendell lab). However, the function of the vast majority of the NORAD sequence, much of which is highly conserved among mammals, remains unknown, and it is unclear if and how it contributes to antagonizing Pumilio activity.

The structures of several lncRNAs were previously interrogated *in vitro* using synthetic refolded RNA ^18–24.^ More recently, transcriptome-wide methods provided additional structural data from within cells. These efforts resulted in important structural and functional insights for several lncRNAs ^25–27^. Yet, while *in vitro* studies cannot reflect the impact of the cellular environment on lncRNA structure, transcriptome-wide studies typically result in low coverage and resolution per transcript. It is increasingly appreciated that inside cells, the structure of RNA is inherently dynamic ^27–33^. This phenomenon is best described for riboswitches, for mRNAs undergoing splicing, and for the genome of several RNA viruses, where structural plasticity increases the functional capacity of the RNA. However, whether mammalian noncoding RNAs adopt alternative conformations during their life cycle and the functional importance of these conformations remains under-explored.

Here we combined *in vivo* structural probing with affinity selection of a single lncRNA, resulting in high-depth and high-resolution maps reflecting the folding of NORAD inside human cells. We reveal that NORAD folds into discrete structural domains and undergoes a structural reorganization in response to certain stress stimuli. NORAD structural domains cluster together the Pumilio binding sites along its sequence and facilitate the ability of NORAD to de-repress Pumilio targets.

## Results

### NORAD structure in unperturbed and stress conditions

In order to probe the RNA structure of NORAD within living cells, we applied the COMRADES method with probes targeting NORAD in HCT116 cells ^31^. Briefly, base-paired RNA was crosslinked inside living cells using Psoralen-TEG-Azide, after which total RNA was extracted, and NORAD was pulled down using a tiling array of antisense biotinylated probes. Following RNA fragmentation, we employed click chemistry to attach biotin to the crosslinked RNA and pulled it down using streptavidin beads. Half of the resulting RNA - the ‘interactions’ sample - was proximity ligated, followed by a reversal of the crosslink and sequencing. In the other half - the ‘control’ sample - the crosslink was first reversed, after which the RNA was proximity-ligated and sequenced.

In order to characterize the structural organization of NORAD in different cellular conditions, we used untreated cells, cells treated with doxorubicin (Doxo), a DNA-damaging reagent previously shown to influence NORAD abundance and subcellular localization ^4,6,7^, and Arsenite (Ar), a reagent that leads to metabolic stress and a strong shift in the localization of NORAD to stress granules ^12,34–36^. Analysis of the sequencing data showed that NORAD reads were enriched more than 1,000 fold following pulldown with biotinylated probes. Approximately 4% of all reads were chimeric, and ~1% represented the base pairing of NORAD inside human cells. In contrast, only <0.1% of the reads in the control samples in which reverse crosslinking was performed before the proximity ligation were NORAD:NORAD chimeras, demonstrating a good signal-to-noise ratio. Overall, the study identified 366,184 base pairing events, which enabled us to build high-resolution structural maps of NORAD inside cells.

**Extended Data Fig. 1:**
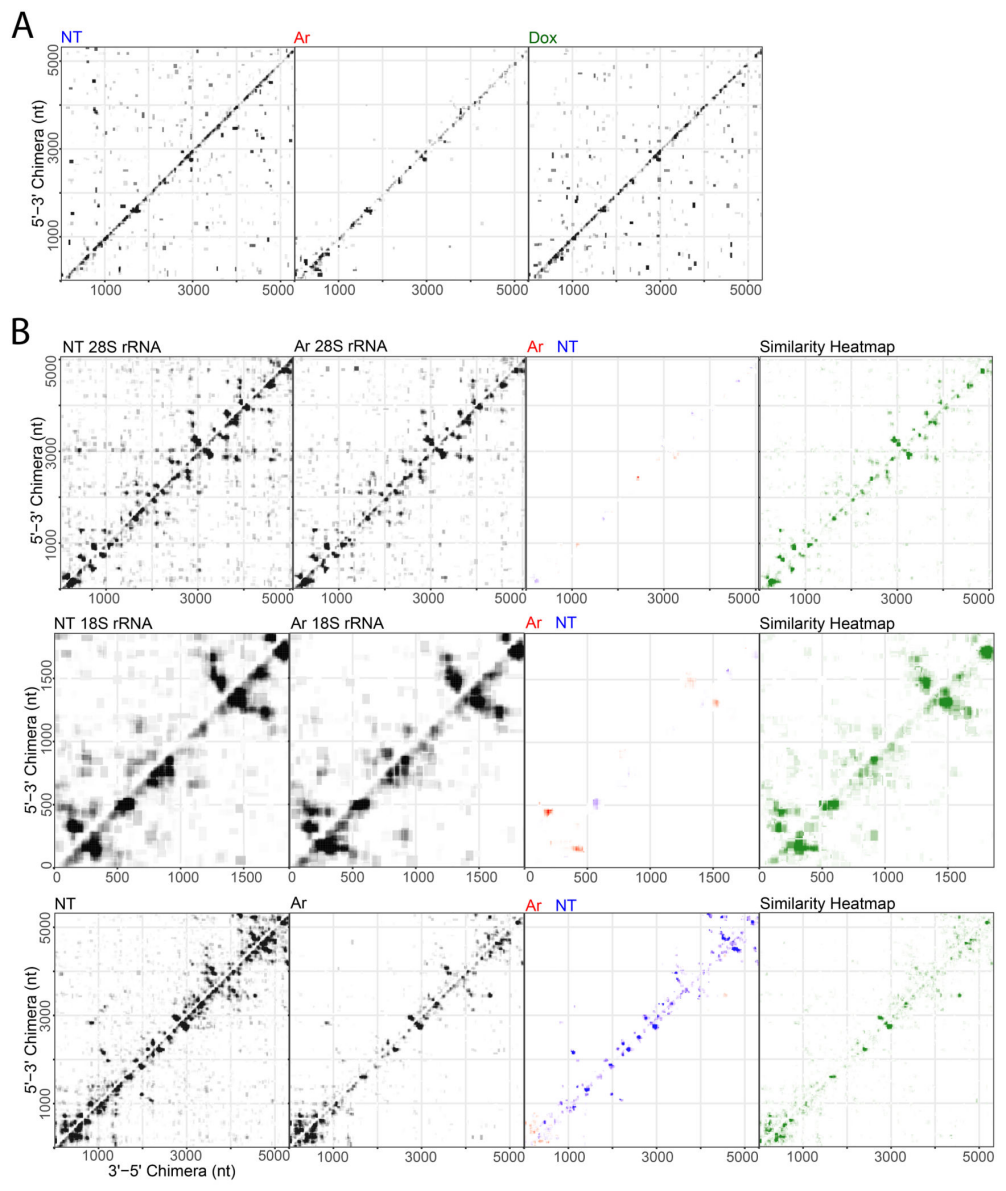


**Figure 1:**
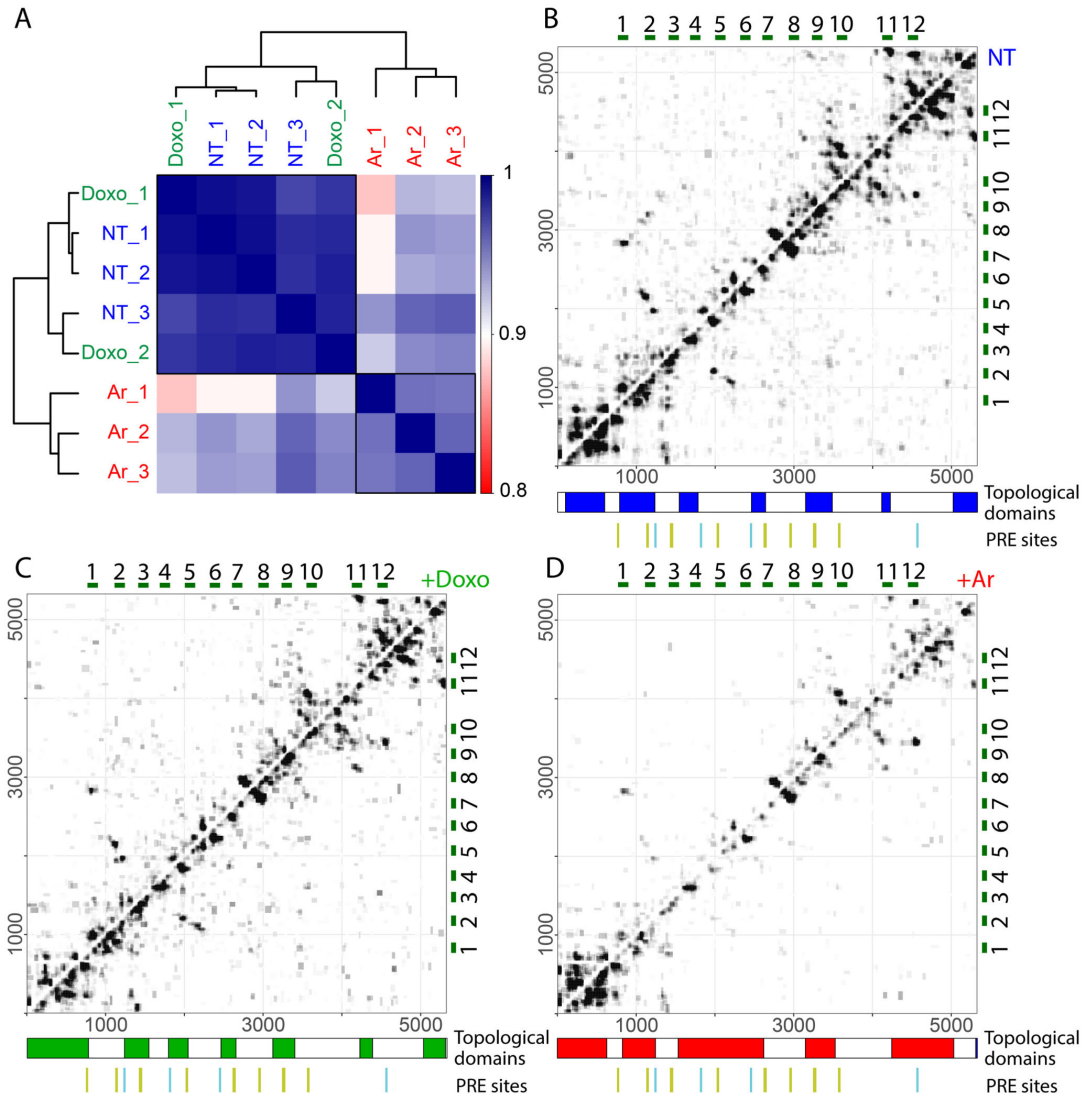
Structural organization of NORAD. **(A)** Pearson correlations between replicate COMRADES experiments in HCT116 cells without treatment (NT_1-3) and treated with doxorubicin (Doxo_1-2) or arsenite (Ar_1-3). Correlations were calculated using counts of NORAD:NORAD chimeras in 100x100-nt windows. **(B-D)** Domain organization of NORAD without treatment (B), and with Doxo (C) or Ar treatment (D). Interaction maps are shown for experiments NT_1 (B), Doxo_1 (C), and Ar_1 (D). The blocks under the heatmaps show the division of NORAD into domains. Domains were called with the program TopDom ^37^ using interaction counts summed across all replicate experiments and a 30-nt window size. The domain analysis was also performed for individual replicates and the results are shown in Table S1. The twelve NRUs are shown on the top and right sides of each heatmap. Canonical PREs found in corresponding positions in the NRUs are marked by vertical lines, with the main PRE clusters in green and four additional PREs in cyan.

We first examined the overall distribution of RNA-RNA interactions within NORAD. Replicates of untreated cells were highly reproducible, similar to those of Doxo-treated cells, and less similar to those in Ar-treated cells (Fig. 1A). Visual examination of the 2D interaction map revealed clusters of intramolecular interactions at the 5’ and 3’ ends of NORAD, with more focal long-distance interactions within the middle part of NORAD which harbors the Pumilio Recognition Elements (PREs). These clusters of intramolecular interactions were not present in control data obtained from cells where crosslinked reversal preceded the proximity ligation (Extended Data Fig. 1A). Furthermore, some of the interactions coincided with the PRE regions (as discussed in detail below).

We next sought to test whether NORAD folds into distinctive structural domains. We applied the TopDom algorithm, originally developed for partitioning genomes into topological domains using chromatin conformation capture datasets ^37^. We identified topological domains within NORAD that were highly concordant between replicates and similar between the untreated and the Doxo-treated cells (Fig. 1B-C). In contrast, Ar treatment led to a substantial reduction of contacts between most NORAD regions, with a notable exception of the 5’ region (Fig. 1D). Taken together, NORAD folds into distinctive spatial domains within cells and undergoes global unfolding throughout most of its sequence in response to arsenite stress.

### Dynamic changes in NORAD structure upon arsenite treatment

**Figure 2:**
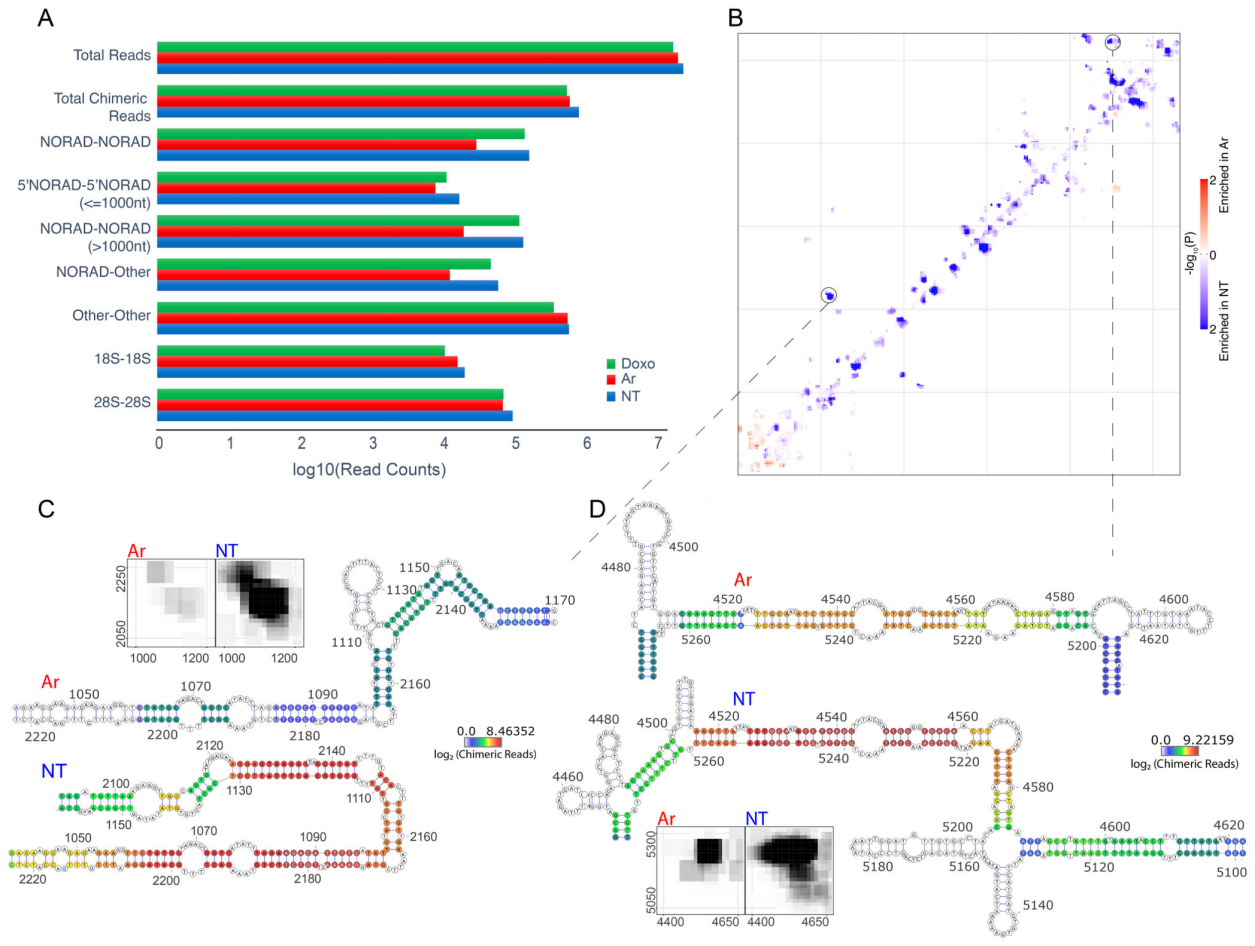
Comparison of NORAD:NORAD interactions between NT, Ar, and Doxo treated cells HCT116 cells. **(A)** Counts of reads summed across all replicates of the indicated COMRADES experiments. The top set of bars shows total counts of non-identical reads; the other sets of bars show counts of non-identical chimeric reads. 5’NORAD-5’NORAD (<=1000nt), chimeras in which both arms are contained within the first 1000 nt of NORAD; NORAD-NORAD (>1000nt), chimeras in which both arms are downstream of the first 1000 nt; NORAD-Other, chimeras between NORAD and another RNA; Other-Other, chimeras between two non-NORAD RNAs, including 18S and 28S rRNAs. **(B)** Differential contact density map with interactions enriched in untreated cells (blue) and interactions enriched in arsenite-treated cells (red). The statistical significance of enrichment in either condition was calculated using DESeq2 in 10-nt windows, corrected for multiple testing. **(C-D)** Example structural changes within NORAD following arsenite treatment. The structures were calculated using the COMRADES pipeline (https://github.com/gkudla/comrades) based on combined experimental data from each treatment. Colours of bases represent counts of chimeric reads supporting each base pair. Insets indicate interaction densities within the corresponding region.

Analysis of chimeric reads revealed a marked reduction of intra-NORAD interactions following arsenite treatment, with a notable exception of the 5’ region, where a substantial number of interactions remained in Ar-treated cells (Fig. 2A). In order to formally test the changes in intra-NORAD interactions upon metabolic stress, we used DESeq2 ^38^ to compare the number of chimeras in windows of 10 nt along NORAD. This analysis showed a significant reduction in contacts throughout NORAD, except the 5’ domain, which was most pronounced within individual NRUs (Fig. 2B). Importantly, no such changes were observed in ribosomal RNAs (Extended Data Fig. 1B). Inspection of the regions with the largest changes showed limited changes in the local structures (Fig. 2C-D), but rather an overall reduction in interaction frequency in Ar-treated cells, suggesting the structure of the central and 3’ part of NORAD becomes globally unfolded upon Ar treatment, rather than adopting a particular alternative fold.

### Spatial clustering of NORAD PREs

**Extended Data Fig. 2:**
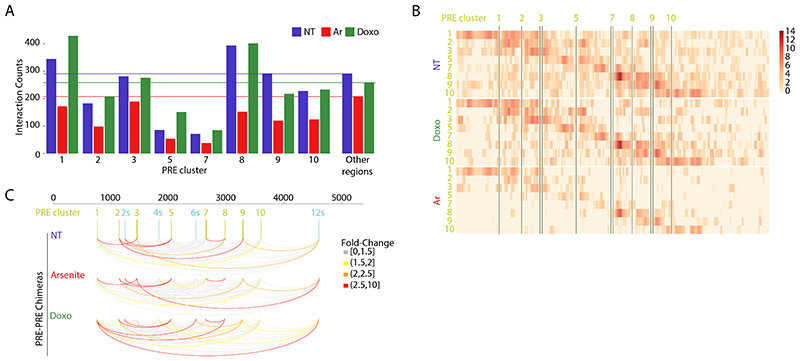


**Figure 3:**
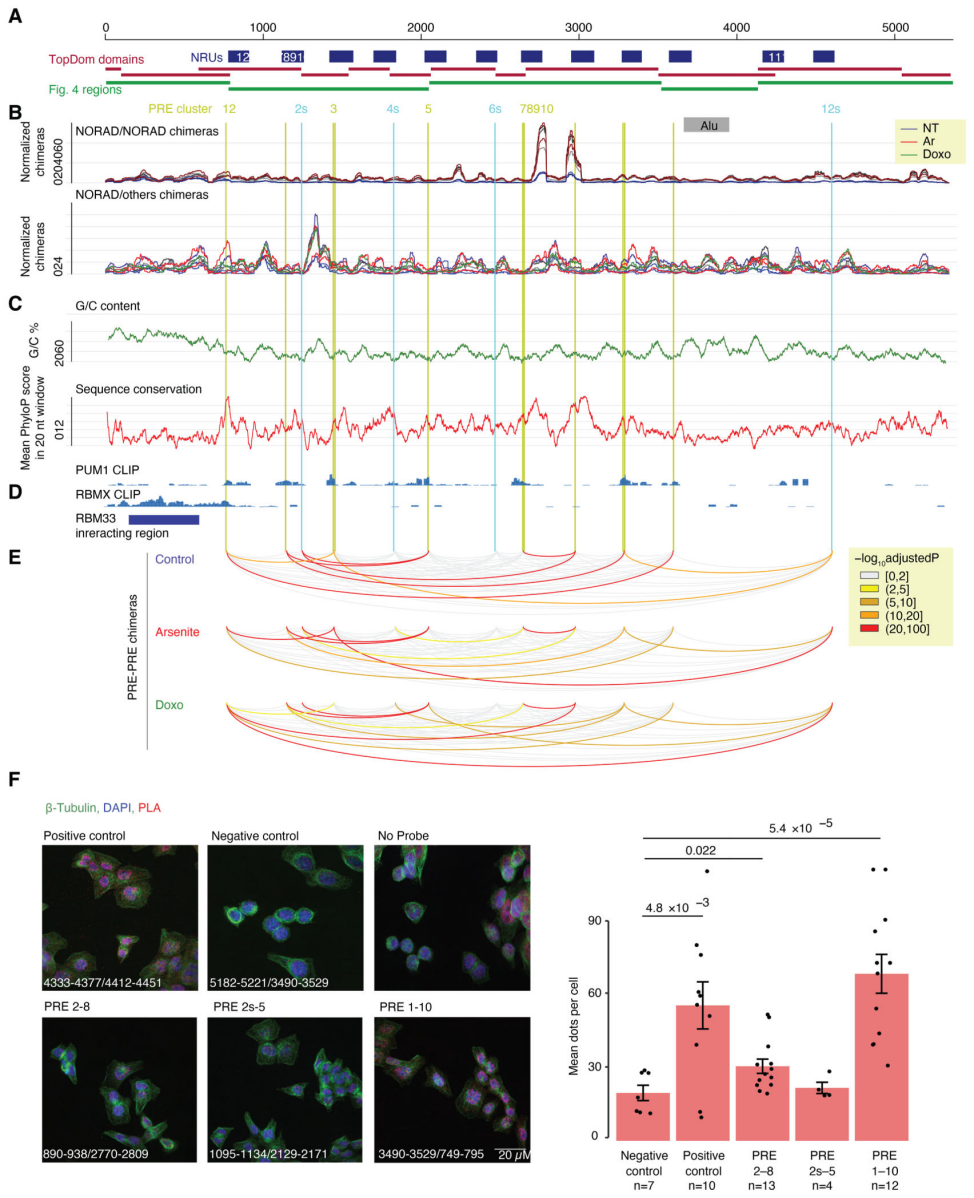
Distribution of intra- and inter-molecular RNA interactions along the NORAD sequence. **(A)** NORAD locus, domains identified when considering all the chimeric reads, and NRUs from ^5^ are demarcated. Canonical PREs found in corresponding positions in the NRUs are marked by vertical lines, with the main PRE clusters in green and four additional PREs (designated as “s” for “supplemental”) in cyan. **(B)** A normalized number of intra-NORAD and NORAD-other chimeric reads is shown separately for each biological replicate with the indicated color code. **(C)** G/C content and PhyloP scores from the 100-way whole-genome alignment from the UCSC browser were computed for windows of 20 bases. **(D)** CLIP data coverage at the bottom for PUM1 and RBMX is from ^6^, and the region with RBM33 CLIP reads is from ^16^. **(E)** NORAD arc diagram connecting pairwise combinations of PRE clusters. Colors are based on - log_10_ of Bonferroni adjusted p-values computed using one-sided t-test. Colored arcs signify a significantly higher number of chimeric reads in PRE-PRE cluster pairs than permuted positions in NORAD. (Arc diagram made using the “R4RNA” R package ^50^. **(F)** RNA-proximity ligation assay. Left: representative images of U2OS cells hybridized with pairs of probes targeting the indicated regions (probe coordinates are listed at the bottom left corner). Imaging was done using 100X oil-immersion objectives (Scale bar 20 μm). Right: Quantification of the mean number of dots per cell in different fields. Results are presented as means ± SEM. The number of cells in each case is shown. P-values computed using two-sided Student’s t-test.

We next combined all the RNA-RNA interactions together and examined the boundaries of the structural domains identified by TopDom and the numbers of inter- and intra-molecular interactions in the context of the 12 NRUs, the 5’ region preceding them, and the 3’ region (Fig. 3A). In the following analysis, we focused on eight canonical “PRE clusters” found in alignable positions in the 5’ part of eight of the NRUs, with each cluster containing one or two PREs. These corresponded to the most prominent peaks in the PUM1 CLIP data from HCT116 cells ^6^ (Fig. 3A). These clusters correspond to 11 of the 15 PREs annotated in ^8^. Four other PREs annotated in that study are in the 3’ parts of NRUs 2, 4, 6, and 8, and have weaker PUM binding in both the CLIP data in that study and in ENCODE data (only the additional PREs in NRUs 4 and 6 show evidence of endogenous PUM binding, Fig. 3D), and we refer to these as “supplementary” PRE clusters “2s”,”4s”, and “6s”. Domains typically contained several NRUs, with two domains corresponding to the 5’ region upstream of the 12 NRUs and two containing the 3’ region downstream of them. Interestingly, regions surrounding the PRE clusters had an overall lower tendency to form interactions with other RNA molecules (Fig. 3B), which was evident for four of the eight PRE clusters in no-treatment and Doxo-treated cells and for all the PREs in arsenite-treated cells (Extended Data Fig. 2A). Upon Arsenite treatment, there was a strong reduction in the normalized number of chimeric reads corresponding to intramolecular NORAD interactions and NORAD interactions with other RNA molecules. This reduction was much less pronounced in the two 5’ domains that are overall much more G/C-rich than the rest of the NORAD sequence (Fig. 3C). Interestingly, the first 1/8 of the NORAD sequence, which was substantially less affected by Arsenite treatment, is also the part that is least capable of recruiting a reporter RNA to stress granules upon arsenite treatment ^12^.

**Extended Data Fig. 3:**
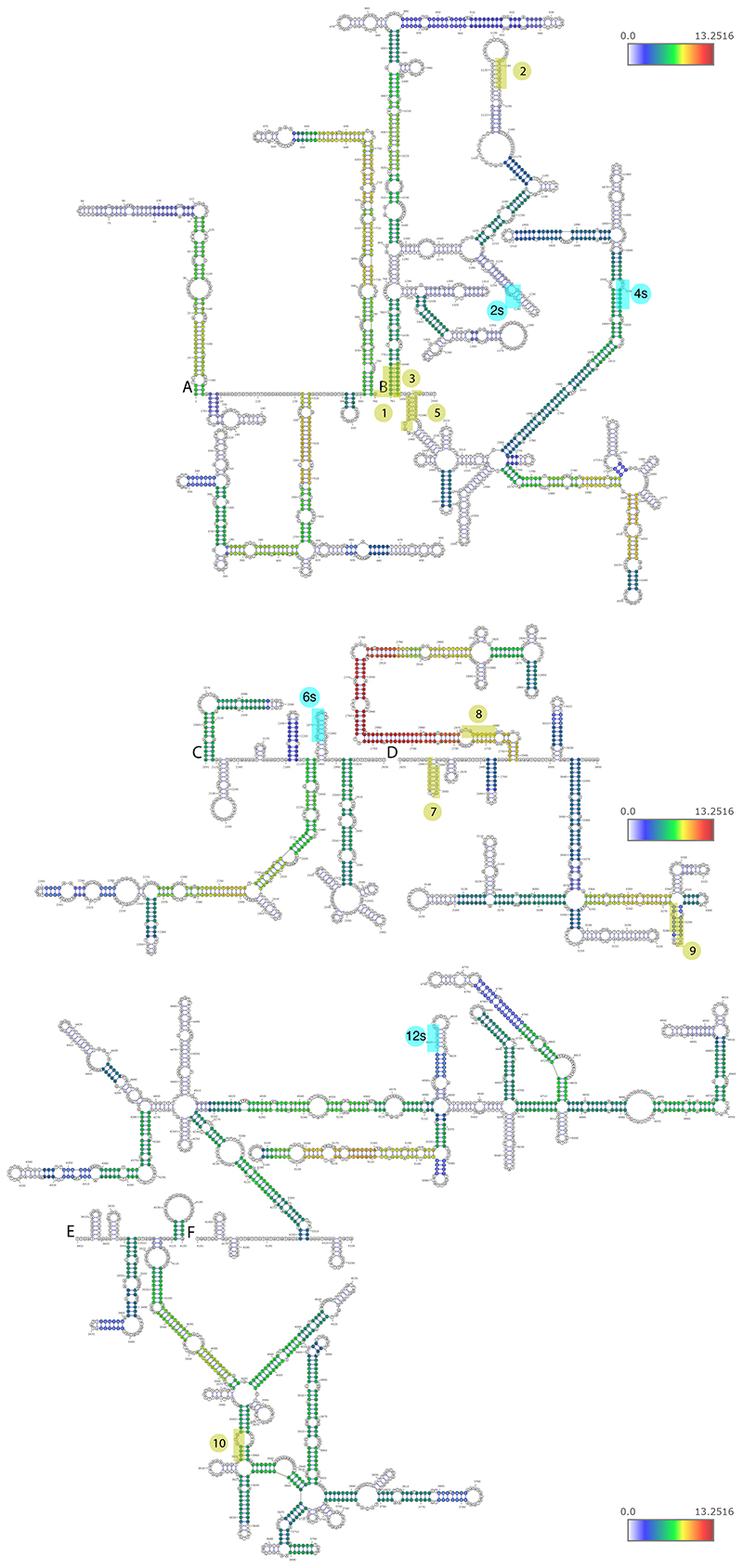


**Extended Data Fig. 4:**
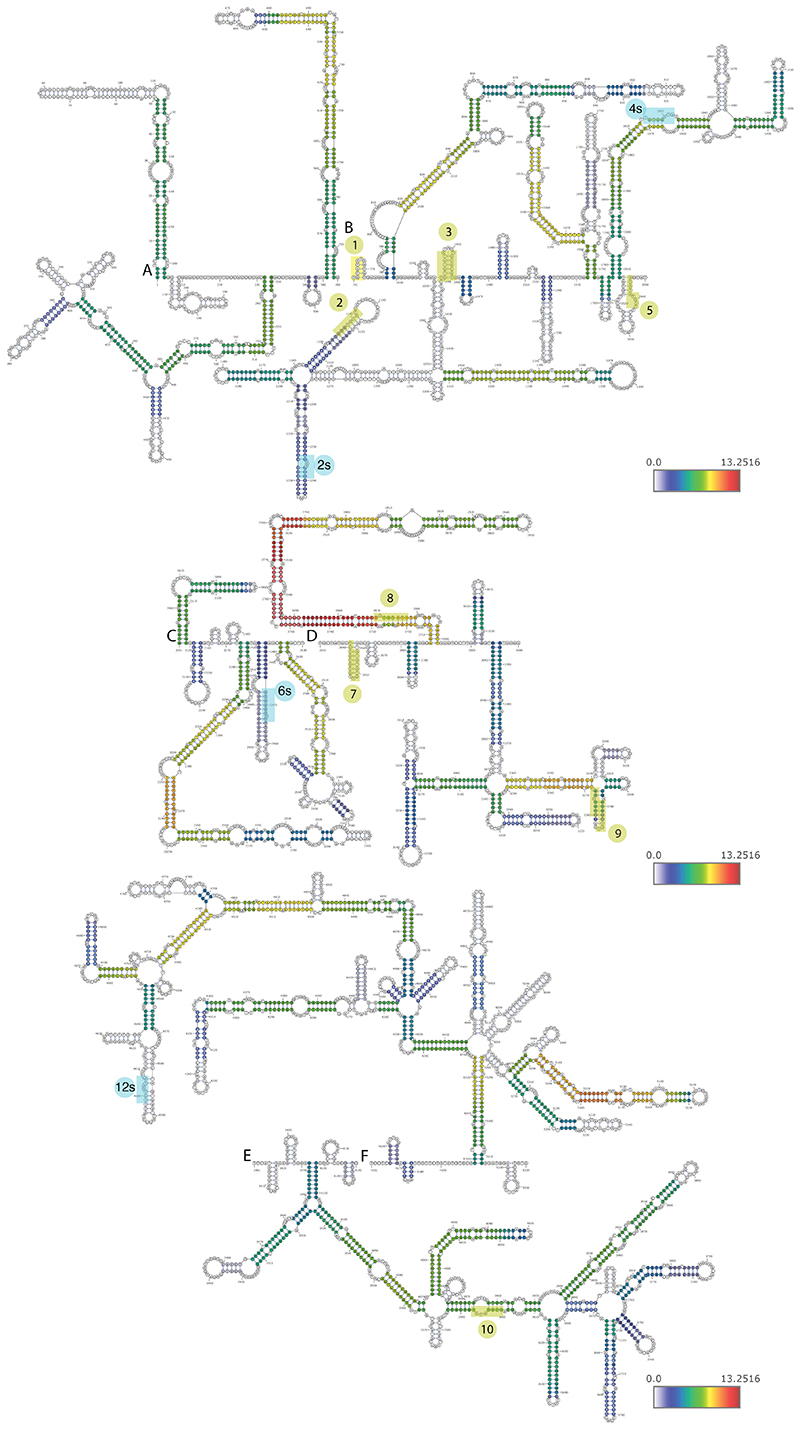


**Figure 4:**
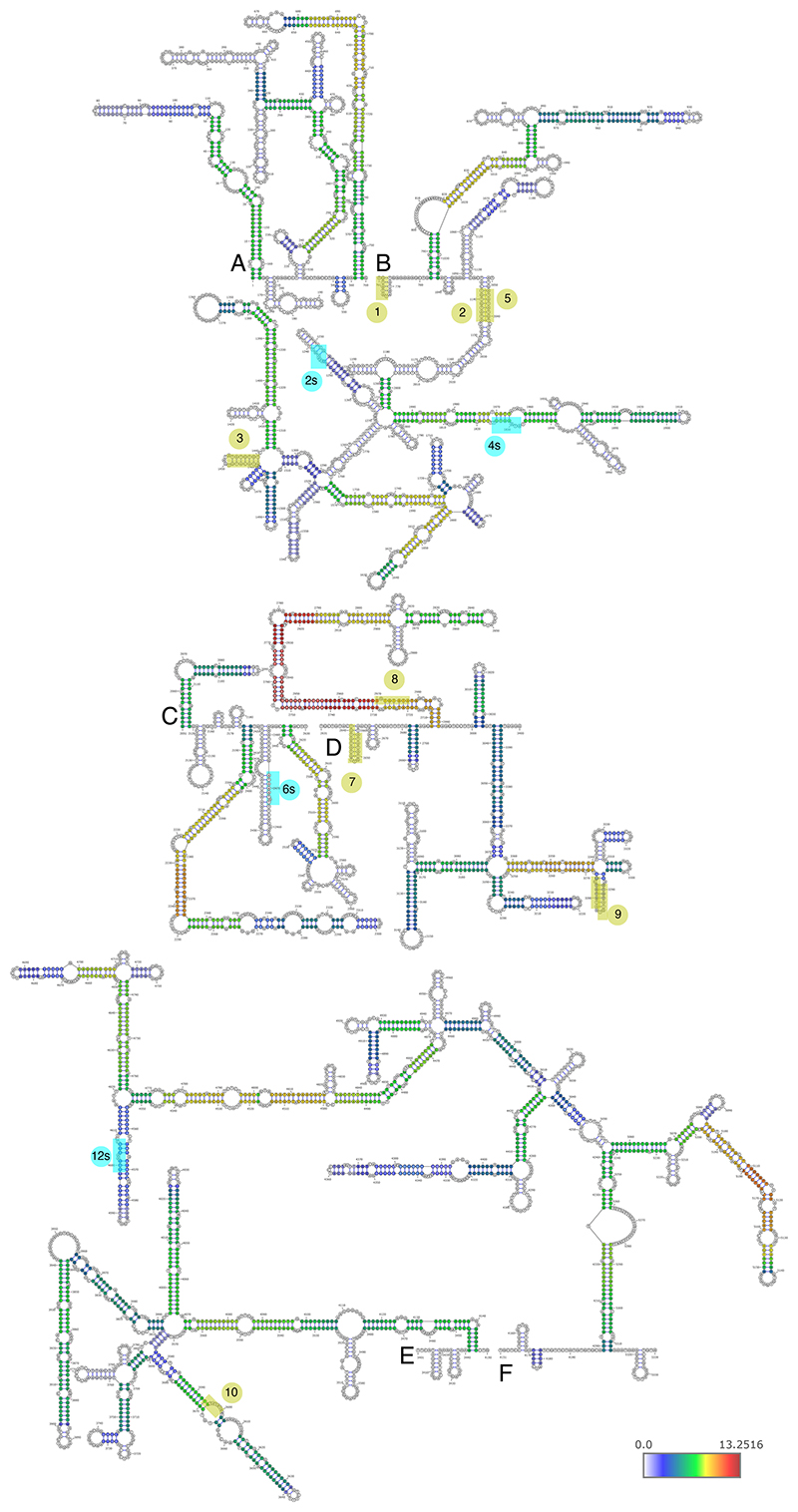
Predicted structures of NORAD domains in untreated cells (NT). Predicted structures of separately folded NORAD regions 1-760 (A), 761-2050 (B), 2051-2630 (C), 2631-3400 (D), 3401-4150 (E), and 4151-5339 (F). Bases are colored based on log_2_ of the number of reads normalized to chimeric reads per million mapped reads. The eight clusters of PREs are shaded and numbered as in Figure 2.

When examining the predicted structures with the strongest experimental support (Fig. 4 and Extended Data Fig. 3-4), we noted that PRE clusters appeared to be in close spatial proximity to other PRE clusters, which was also evident when examining the distribution of chimeras formed by each PRE cluster, an analysis which does not rely on any explicit structure prediction (Extended Data Fig. 2B). In order to formally test whether spatial clustering of PREs takes place, we analyzed the number of chimeric reads connecting regions ±50 nt around the PRE clusters and compared them to random equidistant positions within NORAD. For 8 of the 10 PRE-containing regions, the number of chimeric reads with at least one other region was significantly (P<0.05, adjusted for the number of PRE cluster pairs) larger than expected by chance in all experimental conditions (Table S2, Fig. 3E and Extended Data Fig. 3B). To validate spatial clustering of regions harboring distal PREs, we used a RNA-proximity ligation assay (RNA-PLA ^39,40^) with probes targeting different regions within NORAD, using a pair of probes separated by <50 nt as a positive control, as suggested ^39^ (Fig. 3F). We found a significant co-localization of the regions harboring PREs 1 and 10, separated by ~2,700 nt in the linear NORAD sequence, and to a lesser extent, between PREs 2 and 8, when compared to a negative control region not predicted to interact, between PRE 10 and 3’ end of NORAD (Fig. 3F). Notably, the interaction between the 2s and 5 PREs could was not evident in the RNA-PLA, possible because it occurs more rarely, and so might not be evident in the relatively few cells profiled by RNA-PLA. The overall structure of NORAD thus positions PREs in close spatial proximity to each other in a manner that may facilitate the formation of NORAD-Pumilio bodies ^13^ (see Discussion).

### *NORAD* modular structure contributes to Pumilio antagonism

**Extended Data Fig. 5:**
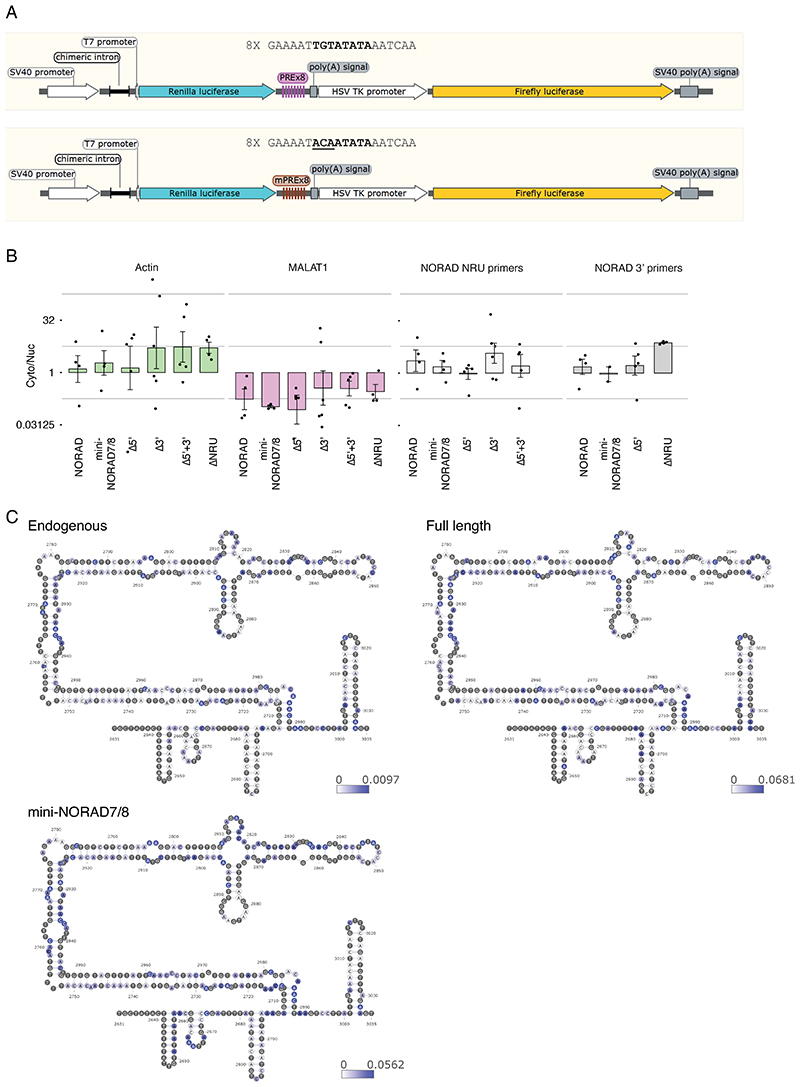


**Figure 5:**
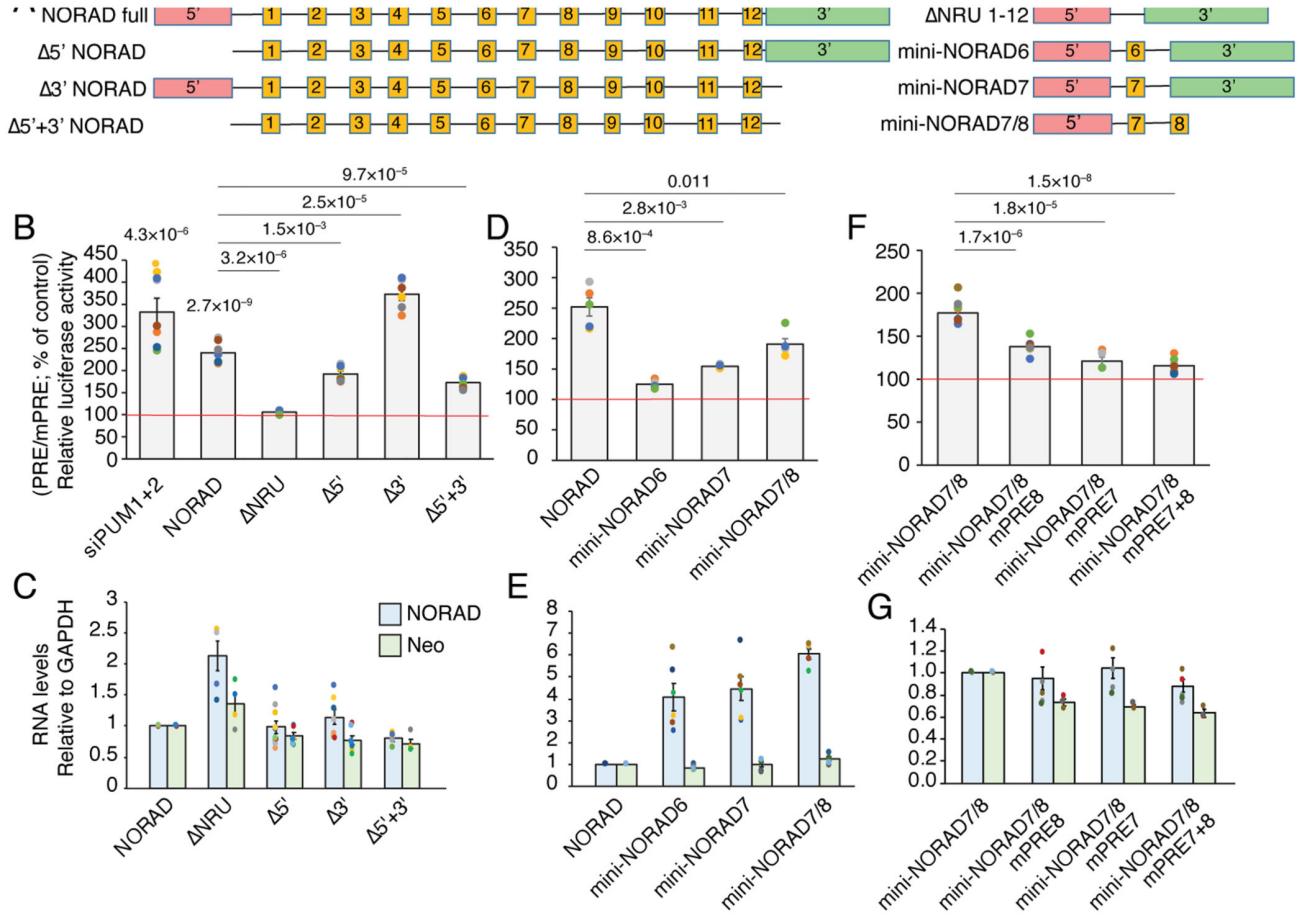
Modular contribution of NORAD sequences to de-repression of a Pumilio sensor. **(A)** Schematic representation of the different NORAD region combinations used for pcDNA3.1-based over-expression in U2OS cells in conjunction with luciferase assays based on 8XPRE and 8XmPRE reporters. **(B)** Normalized luciferase levels in cells co-transfected with 25nM of specific PUM1, PUM2 siRNAs with PRE-reporter (first column) or over-expressing the sequences presented in A. The data are shown as the percentage change from control, where control is designated 100% (scrambled RNA or empty plasmid - red line). n=5–12 independent experiments. P-values for siPUM1+2 and NORAD are for comparison to control cells, two-sided t-test. Other P-values are for the indicated comparisons, two-sided t-test. **(C)** Expression of NORAD variants and of neomycin (Neo) resistance gene expressed from the same plasmid as measured by qRT-PCR. Data are presented relative to the transfection of the plasmid encoding the full-length NORAD and normalized to GAPDH expression levels. n=4–9 independent experiments. **(D-E)** As in B-C for a distinct set of experiments where full-length NORAD or the indicated mini-NORAD versions (D) or mutants (E) were transfected. n=4–6 independent experiments. **(F-G)** As in B-C for a distinct set of experiments where the indicated mini-NORAD versions (F) or mutants (G) were transfected. n=5–8 independent experiments. Results are presented as means ± SEM.

In order to study the contribution of different sequences within NORAD to its ability to inhibit Pumilio activity, we generated a reporter that contains 8 PRE elements within the 3’ UTR of Renilla luciferase (8XPRE). Firefly luciferase, used as a control, was expressed from the same vector under a different promoter. Expression was compared to that of a reporter where all the canonical PRE UGUAUAUA sites were mutated to ACAAUAUA, expected to abolish Pumilio binding (8XmPRE) (Extended Data Fig. 5A), The mPRE control was designed based on a previously described Pumilio reporter ^41^. We have previously used a similar reporter containing 3 PRE elements and showed it to be sensitive to knockdown or repression of PUM1/2 in U2OS cells ^5^, introducing the reporters into cells over-expressing different NORAD variants is an effective and quantitative way to study the efficiency of NORAD sequences in inhibiting Pumilio activity. The 8XPRE reporter was de-repressed by ~4-fold after the combined knockdown of PUM1 and PUM2, allowing for a substantial dynamic range for measurements of Pumilio repression activity (Fig. 5A-B).

We first compared constructs where we removed the 5’ region (bases 1–573, Δ5’ in Fig. 5A), a 3’ region (bases 4682–5343, Δ3’ in Fig. 5A), or the middle part of NORAD (bases 604–4774, ΔNRU in Fig. 5A) which contains all the PRE clusters. Expression of the WT full-length NORAD resulted in ~2.5-fold de-repression of the 8XPRE reporter compared to 8XmPRE (Fig. 5B). As expected, removal of the middle part of NORAD completely abrogated the de-repression (Fig. 5B). Removal of the 5’ region also had a significant effect, whereas removal of the 3’ region led to a significantly stronger de-repression than that caused by the full-length NORAD (Fig. 5B). The combined removal of the 5’ and 3’ modules (Δ5’+3’) led to an effect similar to the removal of the 5’ end (Fig. 5B). Notably, removal of the middle part of NORAD increased expression of NORAD, whereas removal of the other parts did not substantially affect expression (Fig. 5C). We also examined the subcellular localization of the different variants by fractionation of HCT116 *NORAD*^–/–^ cells expressing different variants followed by qRT-PCR. As expected, we found that NORAD lacking the 5’ domain was more nuclear, whereas removal of the middle region containing the NRUs led to increase cytoplasmic expression, presumably due to lack of Pumilio-mediated repression (Extended Data Fig. 5B).

**Extended Data Fig. 6:**
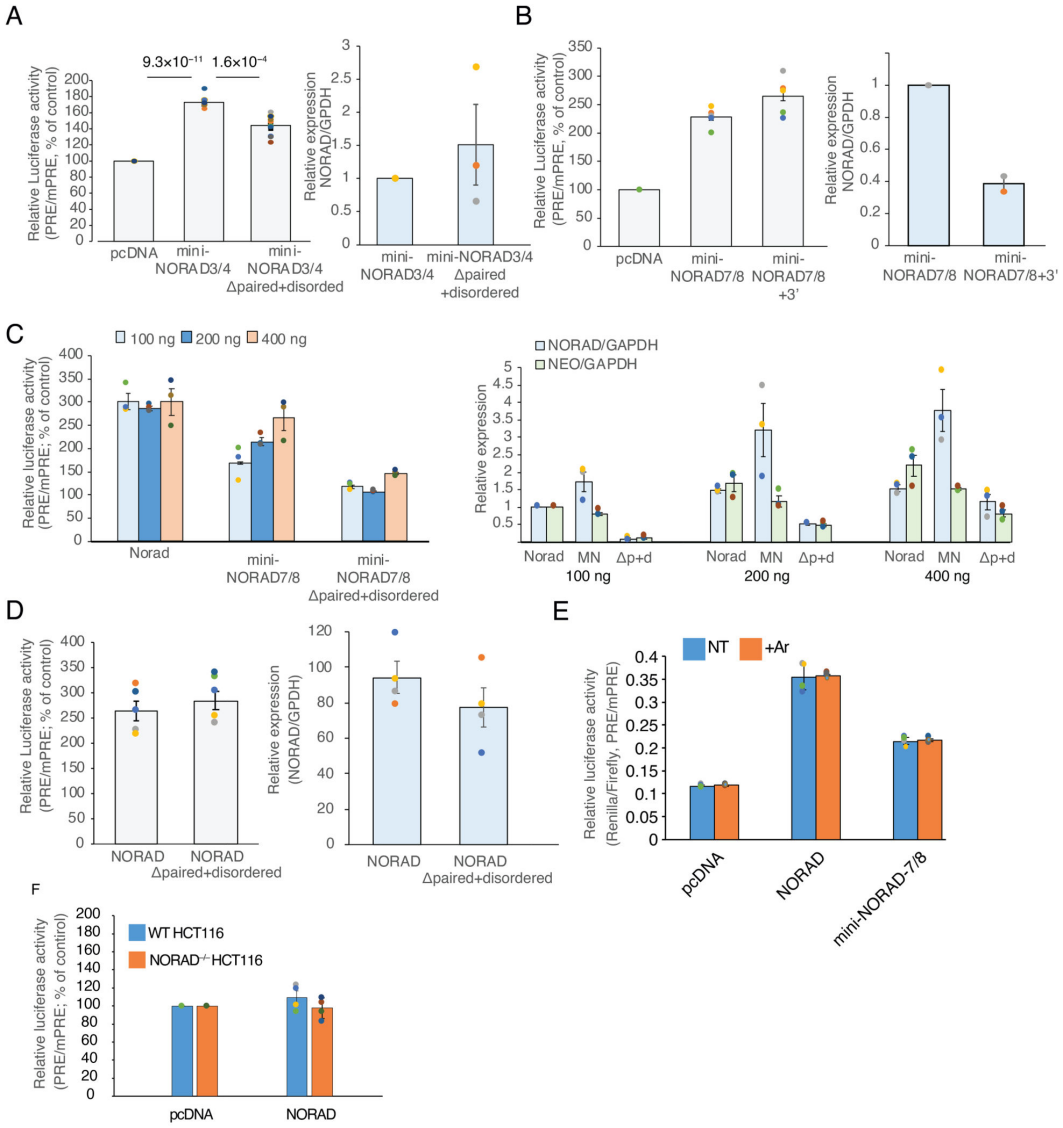


We next tested if a shortened version of the middle part, containing only a subset of the NRUs, is sufficient for de-repression (Fig. 5D). Indeed, a combination of the 5’ and 3’ regions with just one of NRU 6 or 7 had a limited effect on 8XPRE levels (Fig. 5D-E). In contrast, the combination of NRUs 7+8 with only 5’ module (‘mini-NORAD7/8’) was sufficient for potent ~2-fold de-repression of the reporter (Fig. 5D-E), despite having only 1,443 nt of the 5.3 kb in the full NORAD sequence. Notably mini-NORAD7/8 was expressed ~6-fold higher than the full-length NORAD transcript (Fig. 5D). Similarly, a combination of NRUs 3+4 could potently de-repress the 8XPRE reporter (mini-NORAD3/4, Extended Data Fig. 6A). As expected, mini-NORAD7/8 activity was abolished when the three PREs in repeats 7 and 8 were mutated (Fig. 5F-G). Addition of the 3’ module of NORAD to the mini-NORAD7/8 reduced its expression, consistent with the increased expression of the Δ3’ NORAD, but did not substantially affect its ability to de-repress the Pumilio reporter (Extended Data Fig. 6B).

### A structured region between the PREs in repeats 7 and 8

**Figure 6:**
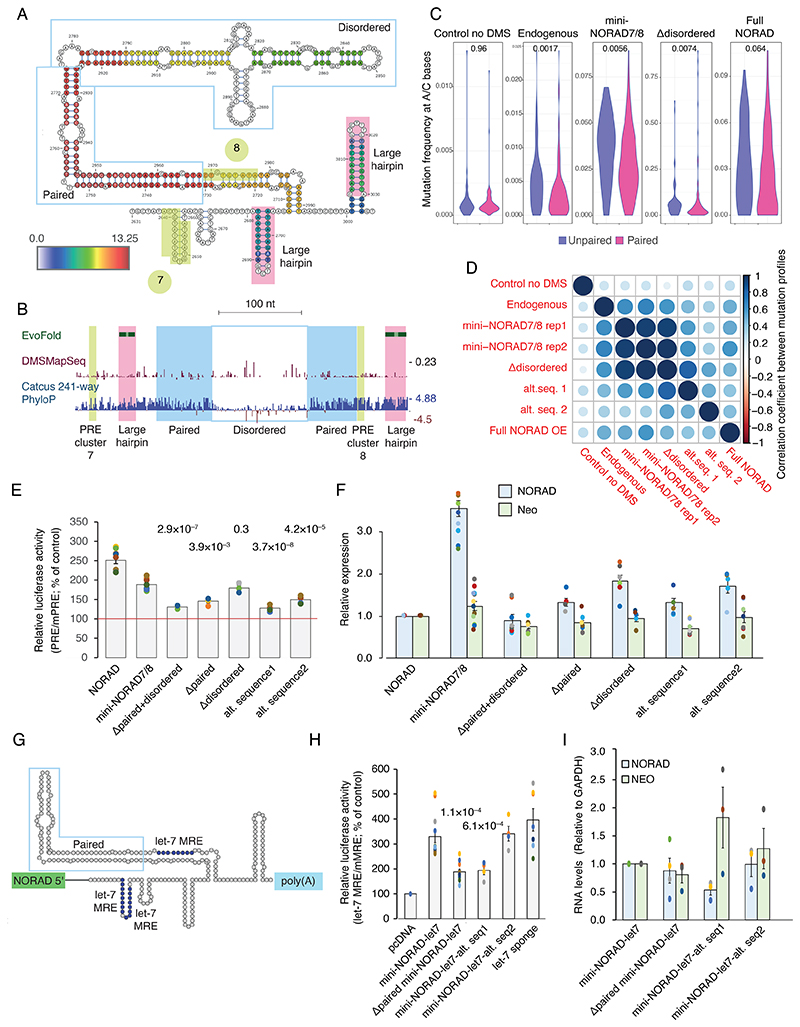
A structured region between PRE clusters 7 and 8 is important for Pumilio de-repression. **(A)** Schematic representation of the predicted fold between PRE clusters 7 and 8, color-coded as in Fig. 3. (B) Structured regions predicted by EvoFold ^51^, DMS-MaPseq reactivity scores ^42^ taken from the RASP database ^52^ and PhyloP scores ^53^ computed based on 241-way multiple sequence alignment, taken from the UCSC genome browser hg38 assembly. **(C)** Mutation frequencies at positions with A or C reference bases and sufficient coverage for the bases predicted to be paired or unpaired in the COMRADES-based secondary structure, in the sequence in A, when probed in the indicated sequences either in WT HCT116 cells (Control and Endogenous) or NORAD^–/–^ cells (mini-NORAD7/8, Δdisordered, Full NORAD). P-values computed using two-sided Wilcoxon rank-sum test. **(D)** Correlation coefficients between the mutation profiles for A/C positions in the region in A for the indicated sequences. **(E)** Normalized luciferase levels in U2OS cells over-expressing mini-NORAD7/8 variants, where the regions indicated in A are deleted or where two alternative sequences with the same predicted fold (alt. sequence 1 and 2) were used. The data are shown as the percentage change from control, where control is designated 100% (empty plasmid - red line). Results are presented as means ± SEM based on at least three independent experiments. Asterisks indicate significant differences from overexpression of mini-NORAD7/8 plasmid; P-values computed using two-sided t-test comparing to to ‘mini-NORAD7/8’. n=3–11 independent experiments. **(F)** Expression of NORAD variants and of neomycin (Neo) resistance gene expressed from the same plasmid as measured by qRT-PCR. Data are presented relative to the transfection of the plasmid encoding the full-length NORAD and normalized to GAPDH expression levels. n=5–12 independent experiments, error bars are ± SEM. **(G)** Schematic view of the mini-NORAD-let7 region where the PRE elements were replaced with let-7 MREs. **(H)** as in E, using HMGA2-based let-7 luciferase reporter vectors in U2OS cells over-expressing the indicated variants of mini-NORAD7/8-let7, a control pcDNA vector, and a let-7 sponge with 6 bulged let-7 sites. n=4–12 independent experiments. P-values computed using two-sided t-test comparing to ‘mini-NORAD-let7’. **(I)** As in F, for the indicated sequences, n=3–4 independent experiments, error bars are ± SEM.

We were next interested in the contribution of the extensively paired regions revealed by COMRADES. The region showing the most extensive intra-molecular pairing within NORAD falls between the PRE clusters in NRUs 7 and 8 (Fig. 1 and Fig. 2), and the predicted fold of the part of NORAD included in mini-NORAD7/8, based on thermodynamics and COMRADES data, suggests that the paired region brings PRE clusters from NRUs 7 and 8 to be in close spatial proximity to each other (Fig. 6A). This paired region is much more conserved in evolution than the ‘disordered’ region between the two paired strands, and the predicted structure is supported by low reactivity in DMS-MaPseq data in HEK 293 cell lines (Fig. 6A-B, data from ^42^). We note that this region is only disordered relatively to the adjacent long paired region.

We first used DMS-MaP-seq to probe the structure of this region in the endogenous *NORAD* in WT HCT116 cells and in exogenous *NORAD* variants in HCT116 *NORAD*^–/–^ cells, where we could distinguish between the endogenous and the exogenous transcripts. As expected, we found higher accessibility of A and C bases in unpaired regions of the predicted structure (Fig. 6C and Extended Data Fig. 5C and Table S3). Replicates of mini-NORAD7/8 transfection experiments were highly similar (Spearman’s R=0.96, Fig. 6D). Folding was similar between the endogenous NORAD and the OE full-length NORAD or mini-NORAD7/8 (Spearman’s R=0.49 and R=0.64, respectively, Fig. 6D).

We next examined the functional contribution of the inter-PRE region in the context of mini-NORAD7/8. Removal of the whole region (‘paired+disordered’) substantially diminished Pumilio de-repression (Fig. 6E), as did independent removal of just the paired region. In contrast, the removal of the ‘disordered’ part had a limited and insignificant effect (Fig. 6E), and as expected had a limited effect on the folding of the region (Spearman’s R=0.96 between mini-NORAD7/8 and mini-NORAD7/8Δdisorded, Fig. 6D). Similar results were obtained in the context of the mini-NORAD3/4, where removal of the paired region reduced the ability to de-repress the luciferase reporter (Extended Data Fig. 6A). We next wondered whether the structure of this region is sufficient for its function. We used RNAinverse ^43^ to design two RNA sequences with the same predicted fold as the structured region but a different sequence and replaced the structure within the context of mini-NORAD7/8. Interestingly, this recoded sequence had a significantly impaired ability to de-repress the reporter (Fig. 6E left), despite similar folding for alternative sequence 1 (R=0.58), and to a lesser extent alternative sequence2 (R=0.32), suggesting that both the structure and the sequence in this region contribute to NORAD function. The changes also affected NORAD expression, and removal of the paired region in particular significantly impaired mini-NORAD expression, as evident when comparing also to the Neomycin resistance gene expressed from the same plasmid (Fig. 6F). We experimented with changing the amount of transfected plasmid, yet the ‘Δpaired’ variants were consistently expressed at lower levels, and so we could not attain comparable expression levels between the different constructs (Extended Data Fig. 6C). Therefore, we conclude that the paired region may assist to stabilize NORAD while bound to Pumilio or may act through another mechanism. Interestingly, when the 7/8 hairpin region was deleted in the context of the full NORAD, it did not affect its ability to de-repress the Pumilio reporter (Extended Data Fig. 6D), which was likely facilitated by the maintained interactions between the other PRE elements (Fig. 3).

### NORAD elements can be co-opted to inhibit other repressors

In order to test if the design principles of NORAD RNA can be utilized to inhibit other RNA binding proteins, we generated a synthetic RNA, mini-NORAD-let7, based on the Δdisordered version of mini-NORAD7/8, in which the three PREs were replaced with three CUACCUCA miRNA response elements (MREs) for let-7 a microRNA that is abundant in U2OS cells ^44^ (Fig. 6G) and known to be functional in these cells ^45^. We profiled the strength of the let-7 repression using a previously described reporter based on the HMGA2 3’UTR harboring seven let-7 binding sites and a mutated 3’UTR as a control ^46,47^. Transfection of mini-NORAD-let7 into cells led to a significant de-repression of the reporter (Fig. 6H). The de-repression was twice stronger when using a vector with the structured element than for one without (Fig. 6H), despite the fact that they were expressed at the same levels (Fig. 6I). This de-repression was comparable to that obtained using a “sponge” vector for GFP (Fig. 6H), which carries six extensively complementary let-7 binding sites (alternating AACUAUACAAGGACUACCUCA and AACUAUACAAUGACUACCUCA, Addgene #29766). Replacing the paired region with one of the the alternative sequences described above, predicted to have a similar fold as the inter-PRE region, significantly reduced de-repression (Fig. 6H-I), consistently with the results in mini-NORAD7/8. These experiments show that the structured elements that support NORAD-mediated repression of Pumilio activity can be utilized to design efficient repressors for other RBPs.

### Intermolecular interactions between NORAD and other RNAs

**Extended Data Fig. 7:**
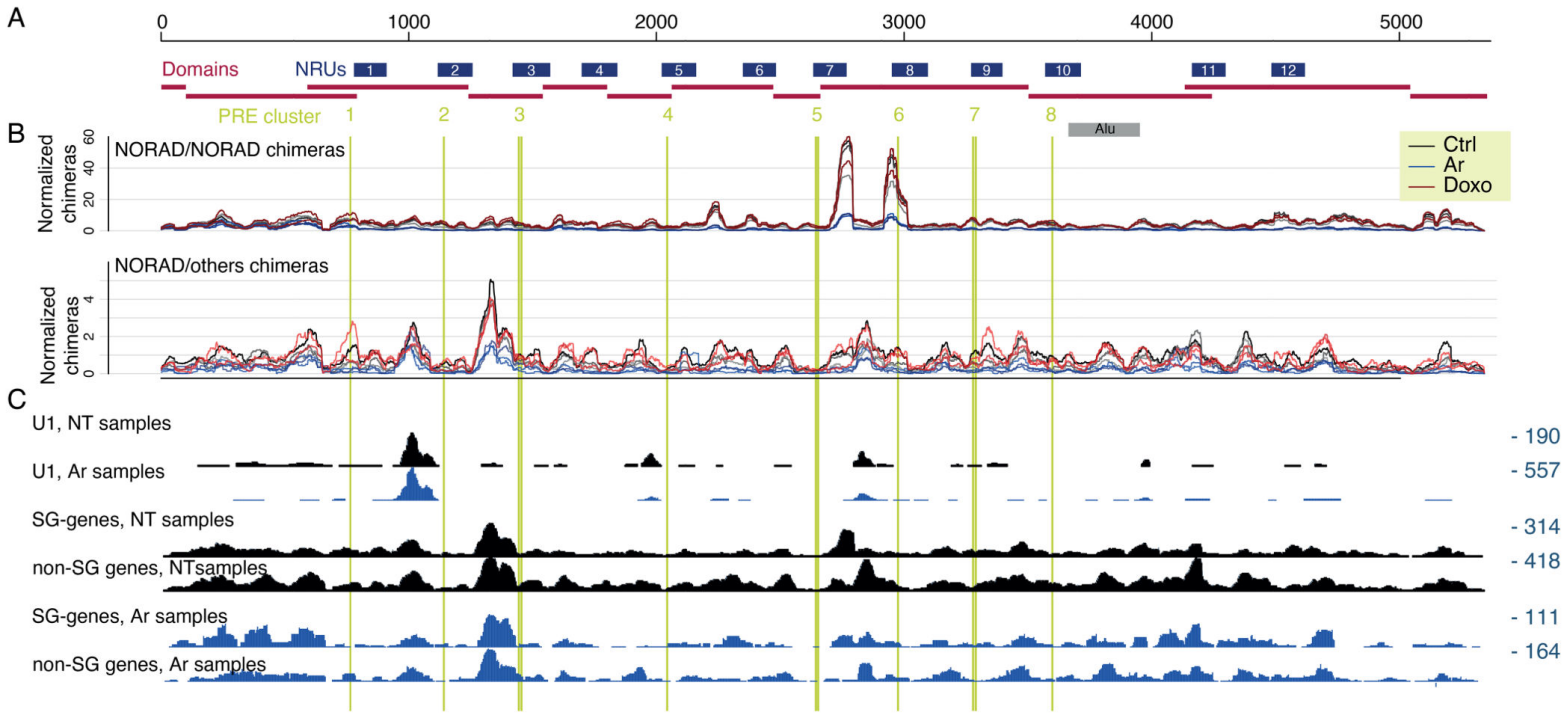


We next focused on RNA-RNA interactions between NORAD and other RNAs. We identified chimeric reads linking NORAD with other RNAs and used DESeq2 ^38^ to evaluate the significance of the enrichment in COMRADES samples compared to the controls in which the psoralen crosslinking was reversed before the proximity ligation (Table S4). Among non-coding RNAs, only U1 snRNA had a significant and reproducible enrichment of interactions with NORAD, most of which were localized in three regions and largely unaffected by the Ar treatment (Extended Data Fig. 7). Interactions with mRNAs appeared in other regions and were spread throughout the NORAD locus with a notable peak between NRUs 2 and 3 and in the ‘disordered’ region between NRUs 7 and 8, and their pattern was also largely unaffected by Ar treatment (Extended Data Fig. 7).

**Figure 7:**
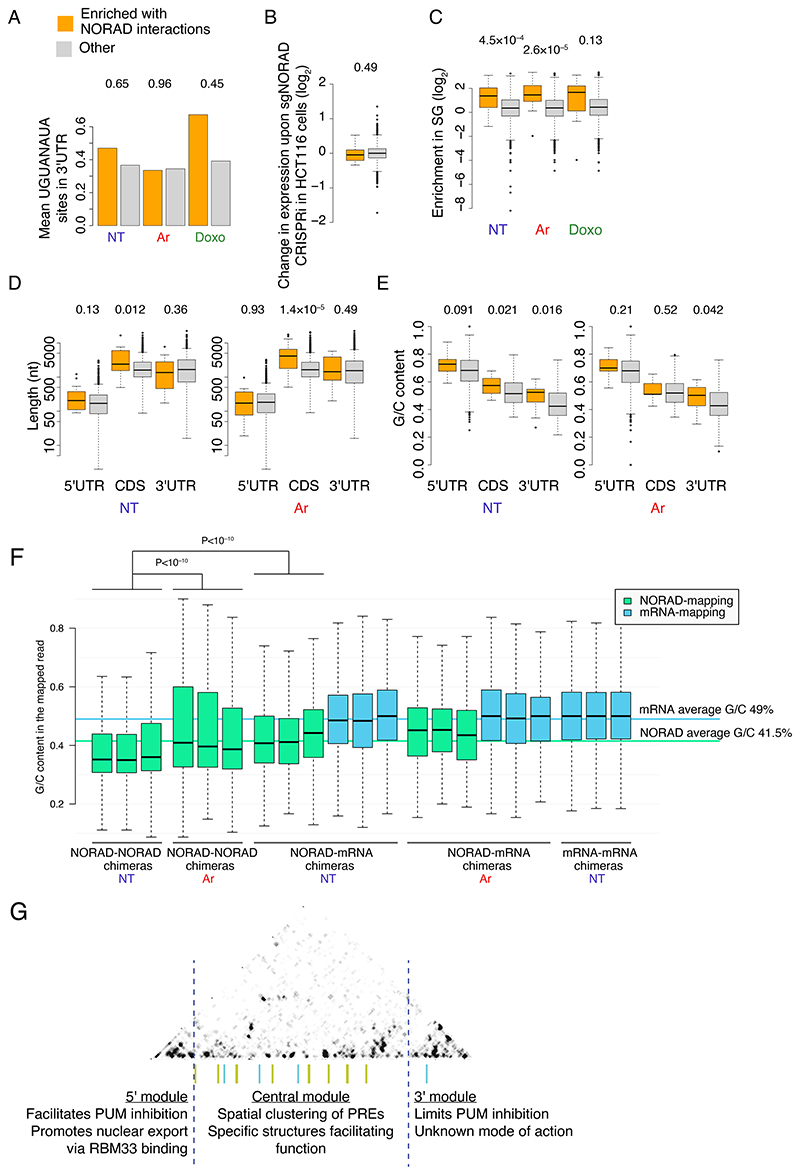
RNA-RNA interactions between NORAD and mRNAs. **(A)** The average number of PREs (defined as UGUANAUA) in the 3’ UTRs of mRNAs enriched in chimeric reads with NORAD in the indicated condition and all other mRNAs. T-test P-values comparing the enriched and the other genes are shown above each condition. **(B)** Changes in gene expression at 96 hr after CRISPRi-mediated KD of NORAD in HCT116 cells (data from ^6^) for genes enriched in interactions (n=18) with NORAD in NT conditions and all other genes with sufficient data (n=1,380). **(C)** As in B for enrichment in the stress granule transcriptome, using data from ^34^. n=18;1,507;18;2,380;9;2021.**(D)** As in B for the length of the indicated mRNA part, for genes enriched in the NT (left) or Ar (right) condition. n=18;1,507;18;2,380;9;2021. **(E)** As in D for the G/C content of the indicated mRNA part (n=19 and n=16 and the enriched mRNAs and n=1,394 and 3,188 for the others). **(F)** G/C content of the part of the read mapped to NORAD or to the mRNAs. Each data point corresponds to a UMI in the sequencing data, and three biological replicates are shown separately in each comparison. Horizontal lines indicate the average G/C content in mRNAs (weighted averages of the 5’UTRs, CDS, and 3’UTRs) and the average G/C content of the NORAD sequence. Comparisons done using Wilcoxon rank-sum test. In **B**-**F** boxplots, the central box shows the interquartile range, and the whiskers extend from the box to the furthest data point within 1.5× the interquartile range and Wilcoxon rank-sum two-sided test P-values comparing the enriched and the other genes are shown above. **G**. Outline of the three major parts of NORAD sequence as revealed by COMRADES analysis. The upper part shows interactions between NORAD regions in untreated cells and it is taken from the heatmap in Fig. 1B. The vertical green and cyan lines show the locations of the PRE clusters. Dashed lines indicate the approximate borders of the 5’ and 3’ modules.

When grouping the chimeric reads by the interacting protein-coding gene, there were 32, 18, and 9 RNAs with a number of chimeras higher in the crosslinked cells compared to their controls in untreated, Ar-treated, and Doxo-treated cells, respectively (fold-enrichment >1.25 and P<0.05, Table S4). Five RNAs were shared between untreated and Ar-treated cells. These transcripts were neither significantly enriched for PREs in their 3’UTRs nor significantly affected by NORAD depletion in HCT116 cells, suggesting that NORAD does not show preferential basepairing with other Pumilio targets (Fig. 7A-B). However, transcripts enriched in each of the conditions were significantly more likely to be enriched in stress granules compared to other genes, with the most significant enrichment observed for transcripts interacting with NORAD in Ar-treated cells (Fig. 7C). Notably, there was no substantial difference in the regions of NORAD enriched with chimeric reads with the 2,462 genes enriched in stress granules (Fold-change >2, P<0.05) vs. regions chimeric with segments other genes (Extended Data Fig. 7), suggesting that there is no particular region in NORAD that preferentially interacts with stress-granule–localized RNAs.

As the stress-granule transcriptome was reported to preferentially include genes with specific characteristics, we analyzed the length and the G/C content of the mRNAs enriched in NORAD interactions and found that they had longer coding sequences (CDS), in particular when considering the enrichments in the Ar conditions, and higher G/C content throughout the RNA (Fig. 7D-E). A longer CDS (but not UTRs) has been previously associated with stress-granule enrichment ^34^. We then wondered if the differences in G/C content might reflect a general tendency for COMRADES to recover interactions of G/C-rich RNAs. To test this, we considered all the chimeric NORAD-NORAD and NORAD-mRNA reads, divided them into the fragments that mapped to NORAD and those that mapped to the mRNA, and computed the respective G/C content of each fragment (Fig. 7F). The G/C content of the mRNA fragments was similar between NORAD-mRNA hybrids and mRNA-mRNA hybrids in an un-enriched HCT116 COMRADES dataset, and matched the average mRNA G/C content, arguing against a strong bias in COMRADES data. Interestingly, within NORAD, in the NT samples, chimeric reads mapping to different NORAD parts were significantly less G/C rich than those mapping to NORAD and mRNAs and below the average G/C content of the NORAD sequence. In Ar-treated cells, NORAD-NORAD chimeras were more G/C-rich, fitting the increase in interactions in the G/C-rich 5’ module and the decline in interactions in the more A/U-rich central part (Fig. 4).

Intramolecular RNA-RNA interactions of NORAD, enriched in the proximity to the A/U-rich PREs and the A/U-rich SAM68 binding sites we described previously ^17^, are thus preferentially formed between more A/U-rich regions of NORAD, whereas the intermolecular interactions, presumably mostly occurring at the “outer surface” of the folded NORAD in the cell, are more G/C-rich, less sensitive to Ar stress, and preferentially connect NORAD with long, G/C-rich RNAs that travel to the stress granules upon Ar treatment.

## Discussion

Our structural analysis indicates that the structure of NORAD is modular, with separate 5’ and 3’ modules and a middle region containing the NRUs, each folded into separate modules (structural domains) (Fig. 7G). Furthermore, long-range interactions within the central region help position the PREs in the NRUs in a closer spatial proximity to each other than expected by chance, with a particularly strong interaction formed by an extensively paired region between the PREs in NRUs 7 and 8. We used these findings to design a ‘mini-NORAD’ gene that can potently de-repress a Pumilio reporter and is thus instrumental for future studies of the ability of NORAD to repress Pumilio activity.

We suggest that the different modules within NORAD contribute to different aspects of its function. The two 5’ modules are substantially more G/C-rich and rapidly evolving than the rest of the NORAD sequence (Fig. 3) and do not contain prominent PREs. The 5’ modules contain the region that binds RBM33 and facilitates NORAD export from the nucleus ^16^, the sequences that were previously shown to be sufficient for an NXF1-dependent export of a single-exon RNA from the nucleus ^15^, as well as a region showing extensive interactions with the RBMX protein ^6^. This region was also shown to be the least effective in recruiting a reporter RNA to stress granules upon Arsenite treatment ^12^, and we see its structure is least affected by Arsenite treatment (Fig. 1). We find that this region is important for the ability of NORAD to inhibit Pumilio repression, and suggest that it likely does so through ensuring efficient export of NORAD to the cytoplasm and/or its stability there, while potentially being responsible for additional functions in the nucleus through its interaction with RBMX.

The functions of the 3’ part of NORAD remain enigmatic. In our experimental setup, we find that it limits the ability of NORAD to inhibit Pumilio function, potentially by limiting its expression. One potential reason could be that without this region, the PREs in NORAD become physically closer to its poly(A) tail, a feature associated with more efficient repression by Pumilio proteins ^48^. It is possible that one function of the 3’ module is thus to provide a “buffer” between the PREs in the NRUs and the poly(A) tail and thus limit the ability of the NORAD PREs to induce its degradation. It is also possible that this region, which contains many bases conserved in evolution, also serves additional, Pumilio-unrelated, functions.

It was recently shown that NORAD nucleates the formation of phase-separated PUM condensates, termed NORAD-PUM (NP) bodies ^13^. Our results complement this model, as they suggest the structure of NORAD helps position some of the PREs in close spatial proximity to each other in a manner that likely increases NP body formation efficiency. For instance, it was demonstrated that four but not two PREs are sufficient for NP formation, but only in a specific context, as mRNA 3’ UTRs with multiple PREs did not efficiently promote NP body formation ^13^. mini-NORAD7/8 contains three spatially co-located PREs and additional U/A-rich sequences that can potentially also bind Pumilio proteins or proteins that can interact with the Pumilio proteins, such as SAM68 ^17^. Single NRUs 7 or 8, with just one or two PREs, do not appear to be sufficient for potent Pumilio de-repression that requires both PRE clusters, as well as the paired structured regions connecting them. The structured regions connecting NORAD PREs thus likely mediate its efficient ability to form NP bodies that are absent in Pumilio-bound multi-PRE 3’ UTRs. Further, NP-bodies were shown to increase in size upon DNA damage induction, and interestingly, we find that the number of significant PRE-PRE interactions becomes higher upon Doxorubicin treatment (Fig. 3).

We report that Ar treatment, which leads to a prominent shift of NORAD localization to stress granules ^12,34,36^, also results in a structural rearrangement within NORAD, which includes a major reduction in intra-molecular contacts within most of NORAD sequence, with a notable exception of the 5’ module, and, to a lesser extent, a reduction in inter-molecular contacts with other RNAs. We cannot exclude the possibility that the stress granule environment is less accessible for the psoralen probing reagent we use, which may underlie some of these changes. Interestingly, Ar treatment overall did not affect the clustering of the PREs within NORAD, which remained significant (Fig. 3B), and correspondingly, NORAD over-expression prior to Ar treatment de-repressed the Pumilio reporter as efficiently as in untreated cells (Extended Data Fig. 6E). The changes in NORAD structure upon Ar treatment thus do not appear to impact the ability of NORAD to inhibit Pumilio but may have other unrelated, stress-granule–related functions. Notably, we did not observe any large-scale changes in the numbers or morphology of stress granules in NORAD-depleted Ar-treated cells.

One limitation of our study is that whereas we characterized NORAD structure in cells where it is expressed endogenously at physiological levels, for the luciferase reporter assays, we use an over-expression setting in WT cells, in which NORAD levels are higher than in the physiological setting. The advantage of this approach is that in U2OS cells, it allows us a large dynamic range of up to ~4-fold de-repression of our sensitive 8XPRE reporter, which is better than what was observed in WT or NORAD^–/–^ HCT116 cells upon NORAD expression (^17^ and Extended Data Fig. 6F). Our experimental model thus allows us to effectively measure differences between the different NORAD variants. Notably, several features that we observed in this setting closely match those observed in a stable expression system, such as the requirement of the canonical PREs for NORAD function and the observation that NRUs 7 and 8 are sufficient for suppressing the chromosomal instability resulting from NORAD loss ^7^.

A recent study has characterized the folding of the NORAD in vitro by the nextPARS approach, which allows measurements of reactivities of individual bases that can be used to inform structure predictions ^49^. This structure is concordant with our finding that NRUs mostly fold independently, with occasional inter-NRU interactions, in particular between NRUs 1–10. When we originally characterized the 12 NRUs and the similarities between them, we noted that several conserved elements, including a small and a larger hairpin, are peculiarly found in some NRUs and not others ^5^. The nextPARS probing data analysis has suggested that, in fact some of the NRUs that do not contain these elements do fold into similar structures some of the time ^49^. Specific proteins that recognize and bind these elements have remained elusive so far. Based on our findings here, a sensible hypothesis is that these small structured elements mainly function in the context of the broader ‘task’ of the overall NORAD structure to spatially position the PREs at favorable distances and orientations relative to one another. This can explain the tolerance of NRUs in evolution, where individual NRUs lost some of the elements that were presumably present in the ancestral NRU prior to its duplication.

## Methods

### Cell culture

HCT116 cells (ATCC) and NORAD^–/–^ HCT116 cells (kind gift from Joshua Mendell) ere cultured in McCoy’s 5a medium supplemented with 10% fetal bovine serum (FBS) and 100 U of penicillin/0.1 mg mL^−1^ streptomycin. U2OS cells (ATCC) (osteosarcoma; obtained from American Type Culture Collection) were routinely cultured in DMEM containing 10% fetal bovine serum (FBS) and 100 U of penicillin/0.1 mg mL^−1^ streptomycin. All cells were maintained at 37°C in a humidified incubator with 5% CO_2_. and were routinely examined to rule out mycoplasma contamination.

### Comrades

The COMRADES method was performed as previously described ^31^. Independent biological replicates were performed using ~150 million cells each. Ar-treated HCT116 cells were supplemented with 0.5 mM sodium arsenite and maintained for 1 hour under growth conditions. Doxo-treated HCT116 cells were supplemented with 1 µM Doxorubicin and were maintained for 24 hours under growth conditions. Cells were washed 3 times with HANKS buffer and were incubated with 0.7 mg/ml Psoralen-triethylene glycol azide (psoralen-TEG azide, Berry & Associates) diluted in PBS and supplemented with OptiMEM I (Gibco) without phenol red for 20 minutes. Subsequently, cells were irradiated with 50 KJ/m^2^ 365 nm UVA on ice using a CL-1000 crosslinker (UVP). Cells were lysed using RNeasy lysis buffer (QIAGEN) supplemented with DTT, and proteins were degraded using proteinase K (NEB). Total cellular RNA was purified using the RNeasy maxi kit (QIAGEN) and quantified using the Qubit RNA BR assay kit.

### NORAD enrichment

RNA was mixed with a tiling array of 50 antisense biotinylated DNA probes, 20 nt each (IDT), targeting human NORAD. RNA was maintained at 37°UnderBardata has been deposited in the GEO database under;C overnight under constant rotation in 500 mM NaCl, 0.7% SDS, 33 mM Tris-Cl pH 7, 0.7 mM EDTA, 10% Formamide. Dynabeads MyOne Streptavidin C1 (Invitrogen) were added, and RNA was maintained for an additional hour rotating at 37°C. Beads were washed 4 times with 2x SSC buffer supplemented with 0.5% SDS and 1 time with 2x SSC buffer without SDS. RNA was released from the beads by degrading the DNA probes using 0.1 units/ml Turbo DNase (Invitrogen) at 37°C for 30 minutes. RNA was cleaned using RNA Clean & Concentrator (Zymo Research) following the manufacturer protocol for capturing RNA bigger than 200 nucleotides.

### Crosslinked RNA enrichment

NORAD-enriched RNA was fragmented by 20 minutes incubation at 37°C with 0.1 units/ml RNase III (Ambion). Reactions were terminated by cleaning RNA with SPRI beads (Amersham) supplemented with Isopropanol. Biotin was attached to cross-linked RNA duplexes by incubating at 37°C for 1.5 h with 150mM Click-IT Biotin DIBO Alkyne (Life technologies) under constant agitation. Following SPRI beads cleanup, biotinylated RNA duplexes were enriched using Dynabeads MyOne Streptavidin C1 (Invitrogen) under the following conditions: 100 mM Tris-Cl pH 7.5, 10 mM EDTA, 1 MNaCl, 0.1% Tween-20, 0.5 unit/ml Superase-In (Invitrogen). Beads were washed 5 times with 100 mM Tris-HCl pH 7.5, 10 mM EDTA, 3.5 M NaCl, 0.1% Tween-20, and RNA was eluted by adding 95% Formamide, 10 mM EDTA solution preheated and incubating at 65°C for 5 minutes. RNA was purified using RNA Clean & Concentrator (Zymo Research). Each RNA sample was split in two: one half was proximity ligated, following UVC irradiation to reverse the crosslink (i.e., interactions sample). The other half was UVC irradiated to reverse the crosslink, and only then was proximity ligated (i.e., control sample). Prior to proximity ligation, RNA was denatured by heating to 90°C and transferring to ice water. Proximity ligation was performed using 1 unit/ml RNA ligase 1 (NEB), 1x RNA ligase buffer, 50mM ATP, 1 unit/ml Superase-in (Invitrogen), at a final volume of 200ul. Reactions were incubated overnight at 16°C and were terminated using RNA Clean & Concentrator (Zymo Research). Crosslink reversal was done by irradiating the RNA with 2.5 KJ/m2 254 nm UVC using a CL-1000 crosslinker (UVP) on ice. Sequencing library preparation was done as described in ^33^. Paired-end 100nt libraries were sequenced using HiSeq 1500 sequencer (Illumina).

### COMRADES data analysis

After obtaining sequencing data in FASTQ format, we removed sequencing adapters (cutadapt -a AGATCGGAAGAGCACACGTCTGAACTCCAGTC -A AGATCGGAAGAGCGTCGTGTAGGGAAAGAGTGT -- minimum-length 10), merged paired-end reads using pear with default settings, collapsed identical reads (bash uniq -c), extracted the 6-nt Unique Molecular Identifier (UMI) from 3’ends of reads, and saved the reads in a deduplicated fasta file, where the number of duplicates and number of UMIs observed for every sequence was encoded in the read ID. To call chimeric reads, we used hyb ^54^ with bowtie2 mapping to a human transcriptome database ^55^ that consisted of spliced mRNAs, tRNAs and other noncoding RNAs, supplemented with the human NORAD sequence (nucleotides 1-5339 from transcript NR_027451.1). We also performed the mapping against a database that consisted of the NORAD sequence alone, which recovered approximately 10% more NORAD:NORAD chimeras, without noticeably altering the pattern of interactions.

We obtained 2M-11M unique mapped reads per replicate experiment, of which 3–4% were chimeric reads; in the untreated and Doxo experiments, 0.8–1.3% of all mapped reads were NORAD:NORAD chimeras, while in Ar experiments, 0.2–0.3% of reads were NORAD:NORAD chimeras. In the control experiments, in which the order of UVC irradiation and proximity ligation was reversed, the fraction of NORAD:NORAD chimeras ranged from 0.02% to 0.12%. Between 3 and 19% of all mapped reads, chimeric or non-chimeric, were mapped to NORAD in the NT, Ar, and Doxo datasets. By contrast, 0.002% of reads were mapped to NORAD in a control COMRADES experiment that did not include NORAD pulldown.

Pearson correlations between replicate experiments were calculated using numbers of NORAD:NORAD chimeras in 100 nt x 100 nt windows, and were plotted in R using the “corrplot” package. The same data was used for the hierarchical clustering of the experiments. 2-dimensional maps of chimera coverage were generated using Java Treeview. To calculate topological domains alongside NORAD, we used the coverage of chimeras in 10 nt x 10 nt windows as an input matrix for TopDom ^37^, and ran TopDom with window.size=30, which resulted in an effective window size of 300 nt. The other TopDom settings were kept as default.

Regions with significantly distinct NORAD:NORAD interactions between the 3 NT datasets and 3 Ar datasets were calculated using DESeq2 based on the numbers of chimeric reads in 10x10 windows ^38^. The direction of change was distinguished using log fold change values, and –log_10_ p-values (capped at 2/–2) were used to plot the interaction heatmap in R, where color intensity reflects statistical significance. Example interactions were isolated and plotted in R with normalization of chimeric counts per million mapped reads, alongside their predicted structures.

Arc plots (Fig. 3) were plotted in R with the “R4RNA” package based on the R-chie web server ^50^. The color of arcs represents the p-values, which were calculated by counting the interactions between two connected PRE sites with a 50nt radius (e.g. between PRE1:710–817 and PRE2:1087–1195), and comparing it to 50nt circular permutations along the genome (e.g. 760–867 and 1137–1195, and so on) using independent t-tests. The resulting p-values were then subjected to Bonferroni correction before plotting.

NORAD secondary structures were predicted using the comradesFold pipeline (Ziv et al. 2018), and the structures were plotted in VARNA. comradesFold generates a set of folding constraints from hyb files, which is then shuffled randomly 1,000 times, and used for structure prediction using hybrid-ss-min. The whole NORAD secondary structure was generated as 6 parts: 1–760, 761–2050, 2051–2630, 2631–3400, 3401–4150, and 4151–5339, each combining domains determined by the TopDom algorithm, with slight adjustments to preserve high confidence structures. Color-coding of base-pairing is based on hyb data-based supporting reads, and was normalized to log2 chimeric counts per million mapped reads.

Numbers of interactions between PRE clusters to other RNA fragments, and non-PRE fragments to other RNA fragments (Figure S3A) were calculated using 50 nt radius for PRE clusters, and 116 randomly generated 110nt-long non-PRE fragments. The average interaction counts for the 116 non-PRE to other RNA fragments were used to provide sufficient data. The counts were also normalized as chimeric reads per million mapped reads.

COMRADES data has been deposited in the GEO database under the GSE188445 accession.

### Real-time PCR analysis of gene expression

Total RNA was isolated from U2OS or HCT116 cells using TRI reagent (MRC), followed by reverse transcription using the qScript Flex cDNA synthesis kit with an equal mix of oligo dT and random primers (QuantaBio 95049), according to the manufacturer’s protocol. Real-time PCRs were performed using the AB quantitative real-time PCR system ViiA 7 (Applied Biosystems). Fast SYBR Green master mix (Life, 4385614) was used for qPCR with gene-specific primers (Table S5). All gene expression levels are presented relative to their relevant control (ΔCt) and normalized to *GAPDH* (ΔΔCt).

### Plasmids and siRNAs

Plasmid transfections were performed in U2OS cells using GenJet In Vitro DNA Transfection Reagent (SignaGen Laboratories). To overexpress NORAD, the pcDNA3.1 vector previously described (Tichon et al. 2016) and available on AddGene (AddGene #120383) was used. Derivatives and mutations of *NORAD* were prepared by Restriction Free cloning ^56^. Primer sequences are given in Table S6. As controls for the overexpression experiments, we used empty pcDNA3.1 (+) vector (Invitrogen). In transfections, 200 ng was used per 30,000 cells in 24-well plates for 48 hr before cells were harvested. For luciferase experiments, we generated vectors with 8 WT PREs and mutated PRE reporters as controls. To construct these reporters, eight wild-types PRE repeats (GAAAATTGTATATAAATCAA) or eight mutated PREs (GAAAATACAATATAAATCAA) were inserted using XhoI and NotI sites in 3’ UTR of Renilla gene in the psiCheck-2 dual luciferase reporter vector (Promega). For let7 reporter, HMGA2 3’UTR harboring seven let-7 binding sites or a mutated 3’UTR ^46,47^ were inserted using NruI and NotI sites in 3’ UTR of Renilla gene in the psiCheck-2 dual luciferase reporter vector (Promega). The reporter in the amount of 25 ng and plasmids with different NORAD variants in the amount of 200 ng were introduced per 30,000 cells in 24-well plates. For co-transfection of siRNAs together with reporters, we used LipoJet In Vitro DNA and siRNA Transfection Kit (SignaGen Laboratories), according to the manufacturer’s protocol. Cells were transfected with 25 nM SMARTpool siRNA (Dharmacon) targeting PUM1 and PUM2 sequences (Table S7) or scrambled siRNA with 25 ng of reporter for 48 hr before harvesting. For the Ar treatment experiment, U2OS cells expressing reporter and NORAD plasmids were treated with 0.5 mM sodium arsenite for an hour before harvesting.

### Luciferase assays

Reporter gene activity was measured in U2OS cells as previously described ^41^. Briefly, 30,000 cells were plated in a 24-well plate. After 24 hr, cells were co-transfected with psiCheck2 plasmids and indicated NORAD plasmids (as described above). Luciferase activity was recorded 48 hr post-transfection using the Dual-Glo Luciferase Assay System (Promega) in the Microplate Luminometer (Veritas). A relative response ratio, from RnLuc signal/ FFLuc signal, was calculated for each sample. The percent of change presented is relative to the control plasmid.

### RNA proximity ligation assay (RNA-PLA)

HCT116 WT cells were plated on sterile 8-well chamber slides for 24 hr. Coverslips were fixed for 10 min with 4% PFA at 20°C–25°C and washed 3 times with 1X PBS. Fixed cells are permeabilized and incubated for 12–18 h with 200nM PLA probes to allow for binding to putative interacting RNAs at 37°C. After 3 washes with 1X PBS the coverslips were incubated with ligation mix for 30 min at 37°C [T4 DNA Ligation Buffer (10×, NEB), PLA Connector oligo (100 μM), PLA linker oligo (100 μM) and T4 DNA Ligase (NEB)]. Rolling circle amplification mix [Phi29 Buffer (10×, NEB), 10 mM dNTPs, BSA (20 mg/mL, NEB), Anti-Amplicon-Cy5 Probe (10 μM) and Phi29 (NEB)] was added for 2 hr at 37°C. Monoclonal Anti-β-Tubulin antibody was produced in mouse, clone TUB 2.1 and used for immunofluorescence with 1:1000 dilution to visualize the cytoplasm alongside DAPI staining (Thermo Fisher Scientific) for the nuclei. Imaging was performed on a Nikon-Ti-E inverted fluorescence microscope with a 100× oil-immersion objective and a Photometrics Pixis 1024 CCD camera using MetaMorph software. Puncta from 5-14 fields were counted using IMARIS (v7.7.2) software.

### Subcellular fractionations

HCT116 NORAD KO cells ^4^ were transfected with *NORAD* constructs using PEI reagent. Cells were collected 48 hr post transfection in PBS, and an aliquot collected as a whole cell extract sample. The reminder of the cells were washed twice in cold PBS and fractionated according to the method described in Lee et. al ^4^. Briefly, cell pellet was resuspended in buffer RLN1 (50 mM Tris-HCl pH 8.0, 140 mM NaCl, 1.5 mM MgCl 2, 0.5% NP-40, 10U/ml RNase inhibitor) and incubated 5 min on ice. The nuclei were isolated by centrifugation and the supernatant kept as the cytoplasmic fraction. Nuclear pellet was washed in 500µl of buffer RLN1 and resuspended in 175µl of buffer RLN2 (50 mM Tris-HCl pH 8.0, 500 mM NaCl, 1.5 mM MgCl 2, 0.5% NP-40, 10U/ml RNase inhibitor). The cytoplasmic fraction was cleared by centrifugation and RNA was extracted using BioTri reagent (Biolab 959758027100).

### DMS-MaP-seq

HCT116 NORAD WT and *NORAD*^–/–^ cells were seeded in a 6-well plate (200,000 cells per well). After 24 hr, *NORAD*^–/–^ cells were transfected with NORAD constructs using PEI reagent. Cells were washed 48 hr post-transfection and incubated with warm DMEM containing 2% DMS for 5 min at 37°C. In parallel, as a control, 2% of DDW was added to another well of cells. Media with DMS was decanted, and cells were washed with 30% of beta-mercaptoethanol solution in PBS. The adherent cells were dislodged using a cell-scraper, and collected by centrifugation at 1000 × g at 4 °C for 5 min. Cells were resuspended in TRI reagent and RNA was isolated according to the manufacturer protocol. Total RNA was DNase-treated for 30 min at 37 °C in 1× TURBO DNase buffer with 1 μl TURBO DNase enzyme (Thermo Fisher Scientific). Reverse transcription was performed in a 10 μl volume with 1 pmol of gene specific primer (Table S8). To begin, a mixture of 3-6 µg of RNA with primer was incubated at 95°C for 30 sec to denature the template, and then it was returned to ice to anneal the primer to the template. To initiate the reaction, 20 U Marathon RT (Kerafast, EYU007) with RT buffer (100 mM Tris-HCl pH 8.3, 400 mM KCl, 2 mM MnCl2, 10 mM DTT, 40% glycerol) and 5 mM dNTPs were added to annealed substrate, and allowed the reaction to proceed for 1 h at 42^0^C ^57,58^.

Two ~ 300 nt amplicons tiled around NRU 7 to 8 were designed to obtain sequencing coverage of the structured region of NORAD (Table S8). Amplicons had an overlapping of 65 nucleotides. Amplicons were generated using Q5 High-Fidelity DNA Polymerase (NEB) with NORAD specific forward and reverse primers, and 2 µl of cDNA per reaction carried out in a 25 µl total volume per amplicon.

The amplicons were cleaned up using Nucleospin Gel and PCR Cleanup Kit (Macherey-Nagel, 740609.50). The purity and size of the DNA bands were visualized by running a 1% agarose gel. Illumina adaptors were added to amplicons by 4 cycles of Q5 PCR. Libraries were cleaned, their concentration and average size were determined by Qubit dsDNA HS Assay Kit (ABP Biosciences) and BioAnalyzer High Sensitivity DNA Analysis (Agilent). Sequencing was done using NovaSeq 600 with 300 nt paired-end reads.

Reads were mapped to the NORAD and alternative sequences using STAR ^59^ and mutations in individual positions in the sequence were quantified using JACUSA2 ^60^.

### Statistical analyses

Data are presented as an average ± SEM; experiments were repeated at least three times. Statistical analyses were performed using Student t-test or one-way ANOVA, followed by Bonferroni multiple comparison tests, when appropriate. In all analyses, a value of P < 0.05 was considered significant.

## Extended Data

## Supplementary Material

Source Data Figs. 1–3 and 5–8 and Extended Data Figs. 2, 5 and 6

Supplementary Tables 1-8

## Data Availability

COMRADES data has been deposited in the GEO database under the GSE188445 accession.
